# Biscrolled Carbon Nanotube Yarn Structured Silver-Zinc Battery

**DOI:** 10.1038/s41598-018-29266-0

**Published:** 2018-07-24

**Authors:** Jae Myeong Lee, Changsoon Choi, Ji Hwan Kim, Mônica Jung de Andrade, Ray H. Baughman, Seon Jeong Kim

**Affiliations:** 1 Center for Self-Powered Actuation, Department of Biomedical Engineering, Hanyang University, Seoul, 04763 Korea; 2 Division of Smart Textile Convergence Research, Daegu Gyeongbuk Institute of Science and Technology (DGIST), Daegu, 42988 Korea; 3 The Alan G. MacDiarmid NanoTech Institute, University of Texas at Dallas, Richardson, TX 75083 USA

## Abstract

Flexible yarn- or fiber-based energy storing devices are attractive because of their small dimension, light weight, and suitability for integration into woven or textile application. Some Li-ion based yarn or fiber batteries were developed due to their performance advantages, realizing highly performing and practically safe wearable battery still remains a challenge. Here, high performance and safe yarn-based battery is demonstrated by embedding active materials into inner structure of yarn and using water based electrolyte. Thanks to biscrolling method, loading level of silver and zinc in yarn electrodes increased up to 99 wt%. Our high loaded Silver and Zinc yarn electrodes enables high linear capacity in liquid electrolyte (0.285 mAh/cm) and solid electrolyte (0.276 mAh/cm), which are significantly higher than previously reported fiber batteries. In additions, due to PVA-KOH based aqueous electrolyte, our yarn battery system is inflammable, non-explosive and safe. Consequently, these high-capacities enable our Silver-Zinc aqueous yarn battery to be applicable to the energy source of portable and wearable electronics like an electric watch.

## Introduction

Flexible yarn- or fiber-based devices are attractive because of their exceptionally small dimensions, light weight, mechanical flexibility, and suitability for integration into woven or textile application, providing advantages over conventional rigid, bulky three- or two-dimensional (3D, 2D) devices^1–3^. Such advantages have inspired a lot of recent researches that have focused on the development of fiber based energy storage device such as supercapacitors^4–9^ and Li-ion batteries^3,10–15^. Realizing highly performing, practically safe wearable battery still remains a challenge. Some progressive researchers have been dedicated to develop yarn or fiber based Li-ion batteries due to their performance advantages such as wide open circuit voltage, high energy density, and low leakage property^16,17^. Unfortunately, from a safety point of view, the Li-ion battery could be an inappropriate system for wearable devices which involves direct contact with human skin due to their explosive, flammable, and toxic materials^17–25^. In this study, we introduce Ag-Zn based alkaline battery system as Li-ion battery as an alternative for safe wearable energy storage application. Unlike organic electrolyte based Li-ion batteries, the present aqueous battery consists of Ag cathode, Zn anode, and water based electrolyte, which are environmentally friendly, safe, and uses relatively non-toxic materials^26–30^. In a technical view, maximizing active material loading level can be a key strategy to achieve a practically high performance battery because the performances normalized by not active material alone but the dimension of entire device (including other cell components like current collector, electrolyte, and separator) can give a practical application picture. Here, in order to achieve a highly performing yarn based alkaline battery, we maximize the specific capacity of the aqueous battery by using biscrolling method^31^ that enables active materials to be loaded onto CNT yarn electrodes with loading level up to 99 wt%. Consequently, the great extents of Ag up to 99 wt% and Zn up to 98 wt% could be loaded onto the CNT yarn electrodes by biscrolling method. Due to high active material loading, Ag-Zn aqueous yarn battery showed high linear capacity in liquid electrolyte (C_L_ = 0.285 mAh/cm) and solid electrolyte (C_L_ = 0.276 mAh/cm), which are significantly higher than previously reported fiber batteries. These high-capacities enable our yarn battery to be applicable to the energy source of portable and wearable electronics like an electric watch.

## Results and Discussion

**Figure 1:**
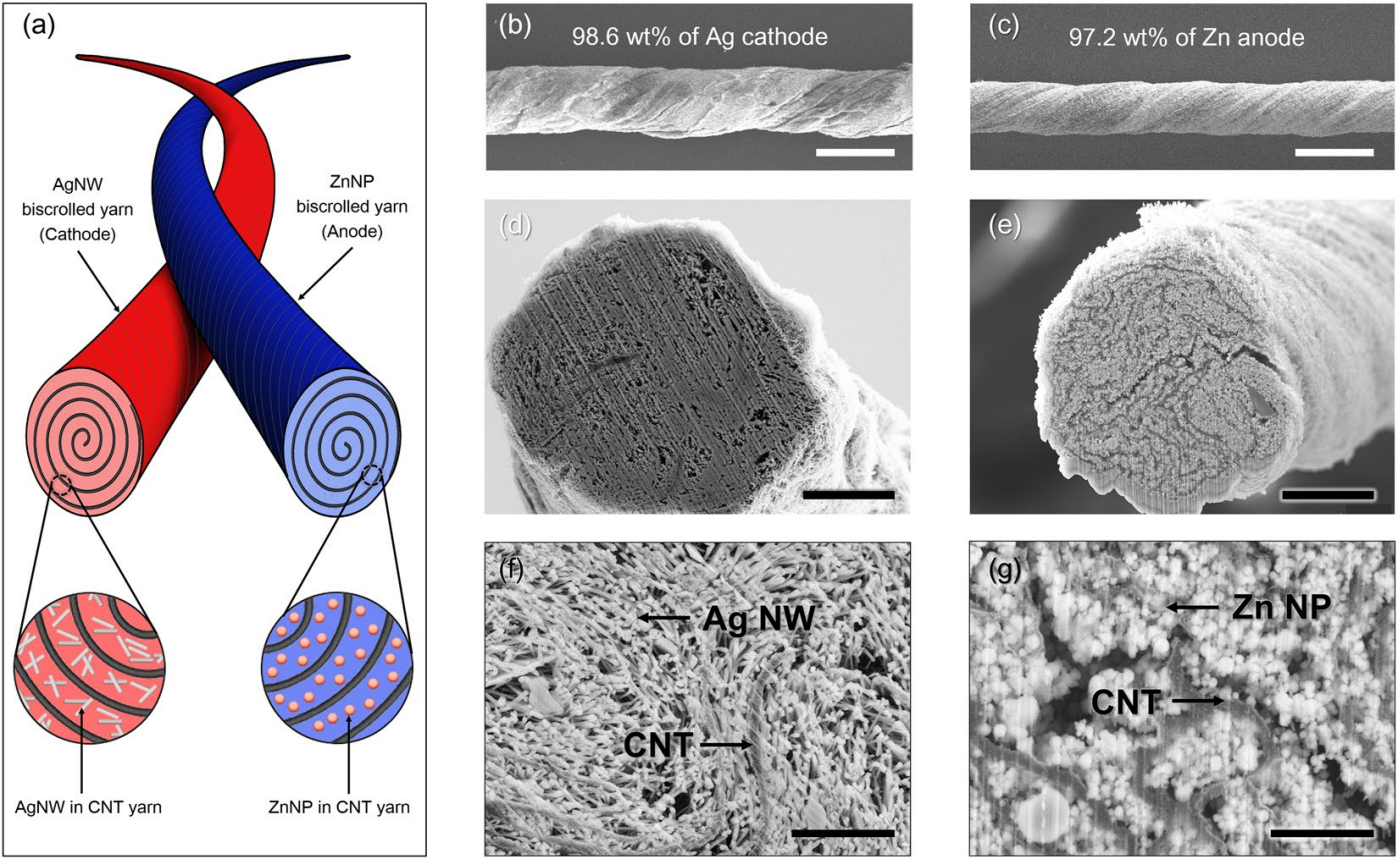
(**a**) Schematic illustration of yarn battery consists of Ag nanowire/CNT and Zn nanoparticle/CNT electrodes. SEM images showing (**b**) the Ag yarn electrode (scale bar = 300 μm), (**c**) the Zn yarn electrode (scale bar = 300 μm), (**d**) the cross-section of the Ag electrode (scale bar = 20 μm), and (**e**) the cross-section of the Zn electrode (scale bar = 20 μm). Magnified SEM images of (**f**) Ag nanowires embedded in CNT (scale bar = 2 μm), and (**g**) Zn nanoparticles embedded in CNT (scale bar = 2 μm).

Schematic illustration for complete aqueous yarn battery that comprises Ag nanowire biscrolled cathode and Zn nanoparticle biscrolled anode is presented in Fig. 1a. Scanning electron microscope (SEM) images for surface of the 98.6 wt% biscrolled Ag nanowires (Ag NWs, 150 nm diameter, 20 μm length) cathode yarn (total mass of Ag was 1071 μg) and 97.2 wt% biscrolled Zn nanoparticles (Zn NPs, 50 nm diameter) anode yarn (total mass of Zn was 541 μg) were shown in Fig. 1b,c, respectively. For structural investigation of biscrolled yarns, the biscrolled yarns were cut using a focused ion beam. SEM micrographs of the cross-sectional area of the 75 μm Ag/CNT yarn showed a porous structure with a porosity around 8.6% and pores smaller than 5 μm (Fig. 1d and Fig. S1a). SEM micrographs and image-based quantitative digital analysis of the cross-section of the Zn/CNT yarn revealed an estimated porosity of about 4% (Fig. 1e and Fig. S2a). EDX mapping and quantitative analyses showed fairly homogeneous distribution of zinc through its diameter (Fig. S2b). It can be confirmed that the aggregated active materials were successfully loaded inside the yarn being confined with CNT scroll galleries. The oxygen was also detected at the EDX mapping (Fig. S2d). It is expected that zinc have been oxidized during the process of dispersing the active material and fabricating the electrode. From its magnifications, the active metal nanoparticles were observed to be well surrounded by adjacent CNT bundles, constructing network structure (Fig. 1f,g). This network structure can provide high electrochemical surface area. Moreover, the functionality of the guest powder can be retained under various mechanical deformations. Due to the metal particles used for active material and MWNT used for current collector, yarn electrodes have good electrical conductivity. The resistance of the 99 wt% silver yarn electrode (diameter = 344.1 µm) increased linearly from 1.2 Ω at 1 cm to 8.2 Ω at 10 cm (Fig. S3a). The resistance of the 98 wt% zinc yarn electrode (diameter = 239.2 µm) also increased linearly from 0.21 kΩ at 1 cm to 2.29 kΩ at 10 cm (Fig. S3b).

The electrochemical reaction of Ag cathode and Zn anode was characterized by the cyclic voltammetry (CV) in 10 mV/s scan rate with three-electrode system with an Ag/AgCl as a reference and a Pt mesh as a counter electrode. 6 M potassium hydrate (KOH) solution was used as an electrolyte. The cathodic reactions exhibits a two-step reactions:

1 $$2{\rm{Ag}}+2{{\rm{OH}}}^{-}\leftrightarrow {{\rm{Ag}}}_{2}{\rm{O}}+{{\rm{H}}}_{2}{\rm{O}}+2{{\rm{e}}}^{-}(0.\,\,46\,{\rm{V}})$$

2 $${{\rm{Ag}}}_{2}{\rm{O}}+2{{\rm{OH}}}^{-}\leftrightarrow 2{\rm{AgO}}+{{\rm{H}}}_{2}{\rm{O}}+2{{\rm{e}}}^{-}\,(0.8\,{\rm{V}})$$

The anodic reaction is:

3 $${\rm{Zn}}+2{{\rm{OH}}}^{-}\leftrightarrow {\rm{Zn}}{({\rm{OH}})}_{2}+2{{\rm{e}}}^{-}\,(1.249\,{\rm{V}})$$

**Figure 2:**
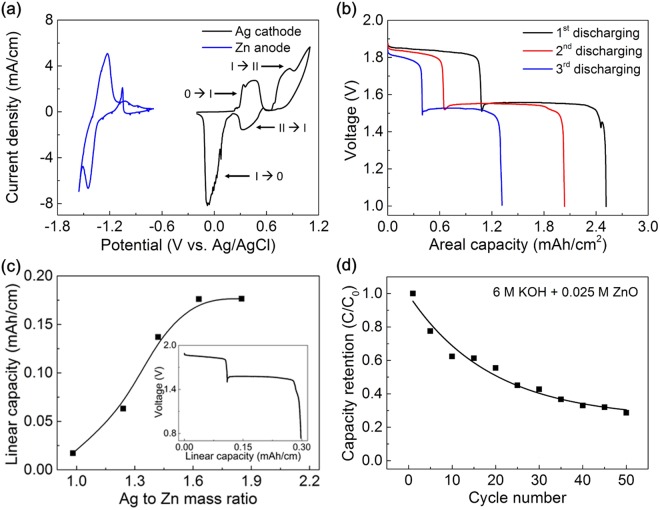
(**a**) CV curves of Ag cathode and Zn anode in 6 M KOH liquid solution measured at scan rate of 10 mV/s. (**b**) Galvanostatic discharge curves of biscrolled Ag-Zn during 3 cycles (**c**) Experimentally derived linear capacity versus Ag to Zn mass ratio (amount of Zn in anode is fixed). Linear capacity is saturated at the point of 1.7: 1 of Ag to Zn mass ratio. (Inset: galvanostatic discharge curves of Ag-Zn yarn battery at current density of 0.1 mA/cm with optimized Ag to Zn mass ratio conditions, 1.7:1). (**d**) Capacity retention during repeated 50 cycles of charge/discharge in 6 M KOH + 0.025 M ZnO liquid solution.

Figure 2a shows the CV curves of Ag cathode and Zn anode. The responses of the cathode and anode are well matched with formation of the Ag and Zn. The full cell reactions of Ag-Zn battery and EMF values are^32^:

4 $${\rm{Zn}}+{\rm{AgO}}\leftrightarrow {{\rm{Ag}}}_{2}{\rm{O}}+\mathrm{ZnO}\,\,(1.86\,{\rm{V}})$$

5 $${\rm{Zn}}+{{\rm{Ag}}}_{2}{\rm{O}}\leftrightarrow 2{\rm{Ag}}+\mathrm{ZnO}\,\,(1.59\,{\rm{V}})$$

The redox peaks in CV curves of Ag-Zn battery system (Fig. S4) showed good agreement with Ag-Zn battery system^29^. The galvanostatic discharge curves of Ag-Zn biscrolled yarn battery during 3 cycles were characterized at current density of 5 mA/cm^2^ (Fig. 2b). Also, according to the two oxidation states of the Ag, two different potential plateaus were clearly observed at 1.45 V and 1.85 V, respectively. Although the reversible reaction between Ag and monovalent Ag_2_O is dominant in electrochemical reaction, the reaction between monovalent Ag_2_O and bivalent AgO contributed to energy storage of our biscrolled yarn battery. Linear capacity versus Ag to Zn mass ratio is plotted in Fig. 2c. The charge storage capability was saturated after the Ag/Zn mass ratio was 1.7. Under optimized Ag/Zn mass ratio, linear capacity of Ag-Zn battery is directly proportional to the weight of active materials and gravimetric capacity retain constant (Fig. S5). Based on this result, we believe that electrolyte penetrated well into the interior of the fiber electrode and all active materials could be in contact with the electrolyte and participate in electrochemical reactions. The highest linear capacity of Ag-Zn biscrolled battery with 1.7 mass ratio was obtained from galvanostatic discharge curve at 0.1 mA/cm as shown in inset of Fig. 2c and found to be 0.285 mAh/cm (wt% of Ag yarn were 99.1 wt% with total mass of 1670 μg and Zn yarn was 98.4 wt% with total mass of 912 μg, respectively). The gravimetric capacity was calculated as 219.3 mAh/g. The linear capacity and discharging voltages are well maintained under current density of 1 to 10 mA/cm (Fig. S6a,b). Figure 2d shows cyclic performance measured during 50 cycles of charging/discharging (capacity from the first discharge was used as a C_0_). As high solubility of zinc in alkaline electrolytes causes shape change and dendritic growth, charge storage capability is degraded by repeated charge/discharge (Fig. S7). In order to improve the cyclic performance, we added 0.025 M ZnO particle into 6 M KOH solution^33^. Thus, the capacity for biscrolled Ag-Zn battery was dramatically improved, retaining about 30% after 50 charge/discharge cycles. In the case of Ag cathode, all peaks in XRD patterns were well-indexed to metal Ag before and after repeated cycles of discharging (Fig. S8a). Nanowires were slightly deformed, but generally maintained their shape (Fig. S8b,c). As for Zn anode, Zn signals were not detected after the repeated cycles in KOH electrolyte (Fig. S9a). Due to the high solubility of zinc oxide in alkaline electrolyte, Zn was rarely observed on the electrode (Fig. S9b). In contrast, Zn electrode discharged in KOH + ZnO electrolyte presented not only Zn but also Zn(OH)_2_ and ZnO signals. Zn dendrite was observed and oxygen elements were detected in SEM and EDX mapping images (Fig. S9c). Although the cyclic performance could be increased, it is evident it needs to be studied through further studies.

**Figure 3:**
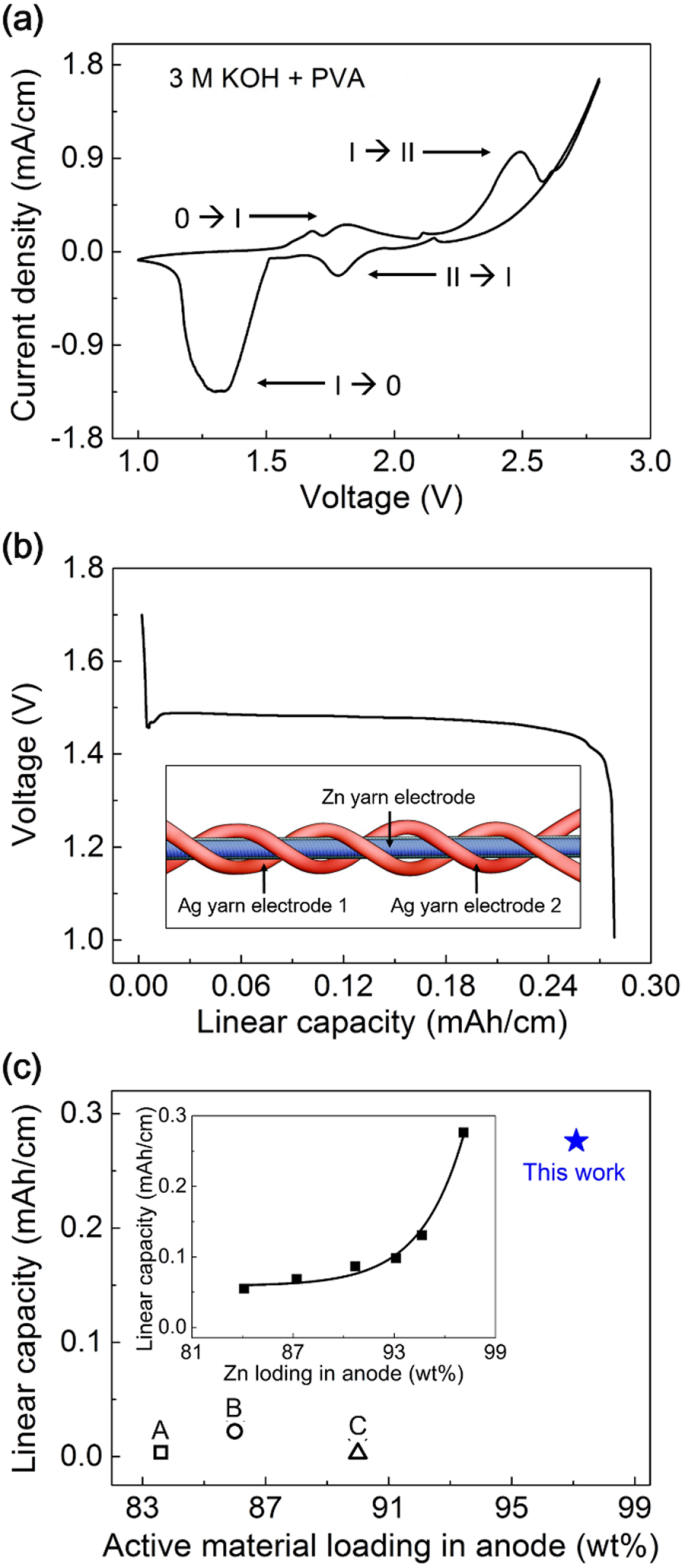
(**a**) CV curve of Ag-Zn yarn battery in 3 M KOH + PVA solid electrolyte measured at scan rate of 10 mV/s. (**b**) Galvanostatic discharge curves of biscrolled Ag-Zn yarn battery. (inset: schematic illustration of 3-plied Ag-Zn yarn battery) (**c**) Linear capacity comparison with present fiber type battery: (A) LTO and LMO winding fiber battery^11^, (B) LTO and LMO coil battery^12^, and (C) LTO and LMO two-plied fiber battery^14^ versus wt% of electrode. Inset shows the linear capacity as a function of Zn loading in anode (the Ag to Zn mass ratio is fixed with 1.7: 1). The highest value of active material loading in anode and linear capacity is 98.1 wt% and 0.276 mAh/cm, respectively.

As demonstration of solid-state energy storage system is an interesting issue for wearable application, the Ag-Zn battery cell with solid gel electrolyte was prepared. Since solid gel electrolyte serves both as electrolyte and separator, it must be provide electrical insulation, exchanging of reactants, electrochemical and mechanical stability during various mechanical deformations. Various type of electrolytes such as hydrogel, polymer or ionomer have been studied and further researches are required to develop the fiber based batteries to the real applications. The hydrogel electrolytes including polyvinyl alcohol (PVA) and KOH is most widely used alkaline solid gel electrolyte due to their high ionic conductivity, mechanical properties, and holding the electrolyte while maintaining flexibility^34–36^. 3 M KOH with 10 wt% PVA was used in these research. CV curve measured at 10 mV/s for gel electrolyte coated two electrode configuration based yarn battery is shown in Fig. 3a. As in the previous measurement in liquid electrolyte, two distinctive oxidation and reduction peaks were observed. Because the optimized mass ratio of Ag/Zn is about 1.7, the Ag electrode is much thicker than the Zn electrode. Here, we divide thick Ag electrode into two electrodes. Two Ag biscrolled cathode yarns and PVA coated single Zn biscrolled anode yarn were plied and the three plied yarn electrodes were coated by PVA-KOH gel electrolyte to assemble the complete solid-state yarn battery (Fig. S10). Schematic illustration of 3-plied yarn battery is shown in inset of Fig. 3b. Although using full voltage range of the Ag-Zn battery gives higher capacity and energy density, efficiency and stability of battery were also regarded as other important factors^37^. Here, using PVA-KOH electrolyte, electrochemical performances of battery were measured between 1 and 1.8 V. The linear capacity of the biscrolled structured silver-zinc aqueous yarn battery calculated from the galvanostatic discharge curve were 0.276 mAh/cm (Fig. 3b), which can be converted into 116.5 mAh/g (diameters of Ag yarns were 343.3 and 357.8 µm and Zn yarn was 220.2 µm, respectively) based on the total weight of MWNT and active materials. Active material loading in anode and linear capacity of our silver-zinc aqueous yarn batteries are shown and are compared with other flexible or stretchable fiber based batteries in Fig. 3c. The active material loadings in the cathode were 98.6 and 98.7 wt% (752 μg and 804 μg of Ag, respectively) and anode was 98.1 wt% (842 μg of Zn) and the highest values of the length, areal, and volume-normalized specific capacities (denoted as C_L_, C_A_, C_V_) in solid gel electrolyte for our yarn battery are 0.276 mAh/cm, 0.93 mAh/cm^2^, and 111.3 mAh/cm^3^, respectively, at a discharge current of 0.1 mA/cm, where the dimensions of the total active materials (presently the Ag nanowire/CNT and Zn nanoparticle/CNT biscrolled electrodes) were used for normalization. Especially, the active material loading in the electrode and length capacity of our yarn battery is the best among yarn or fiber based batteries ever reported^11,12,14^. For example, the length and areal capacities of our fiber battery are one or three orders of magnitude higher than previous yarn type of batteries that used Li_4_Ti_5_O_12_ (LTO) and LiMn_2_O_4_ (LMO) with winding around stretchable core fiber (active material loading: 57.7 wt% in cathode and 83.6 wt% in anode, C_L_: 0.0036 mAh/cm)^11^ or LTO and LMO with coiled structure (active material loading: 65 wt% in cathode and 86 wt% in anode, C_L_: 0.022 mAh/cm)^12^ or LTO and LMO with two plied fiber (active material loading: 78 wt% in cathode and 90 wt% in anode, C_L_: 0.0028 mAh/cm)^14^. Areal and volumetric capacities are compared with that of other fiber batteries in Table 1. Our Ag-Zn fiber battery resulted better areal and volumetric capacities.

**Table 1:** Performance comparison table showing active mateiral loading weight and linear, areal, volumetric, and gravimetric capacities.

	Fiber or yarn battery (Ref. No.)	Active material load (wt%)	C_L_ (mAh/cm)	C_A_ (mAh/cm^2^)	C_V_ (mAh/cm^3^)	C_g_ (mAh/g)
This work	Ag-Zn with 6 M KOH (liquid)	99.1/98.4	0.285	2.59	256.4	219.3
	Ag-Zn with 3 M KOH + PVA (solid)	98.6, 98.7/98.1	0.276	0.93	111.3	116.5
Li-based fiber battery	CNT.MnO_2_ – Li [10]	4.1/X (metal wire)	—	—	109.62	218.3
	CNT/LTO – CNT/LMO [11]	57.7/83.6	0.0036	0.00743	0.248	91.3
	CNT/LTO – CNT/LMO [12]	65/86	0.022	0.438	109.42	92.4
	CNT/LTO – CNT/LMO [14]	78/90	0.0028	0.00458	0.073	150
	Polyimide/CNT – LMO [15]	51/49	—	—	—	123

**Figure 4:**
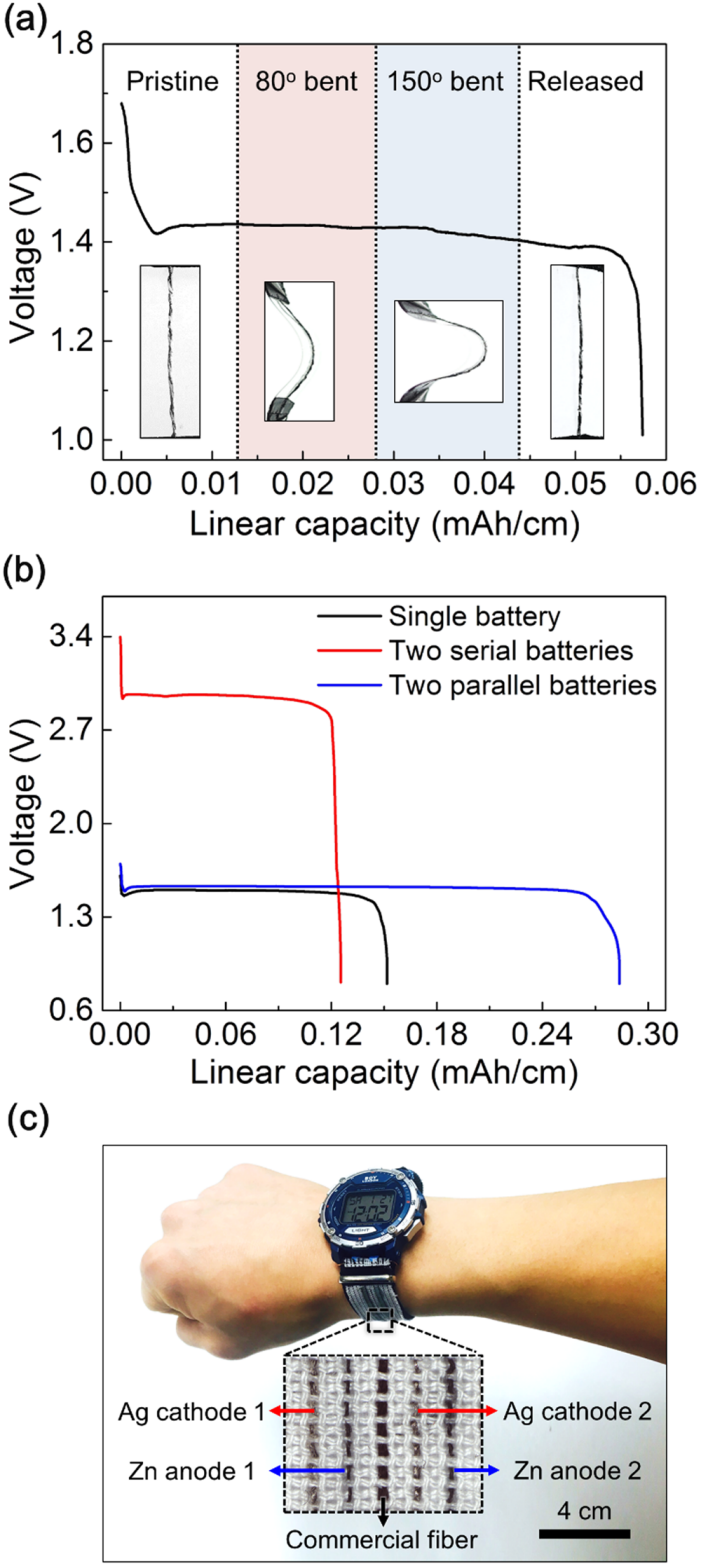
Application. Galvanostatic discharge curves of (**a**) Ag-Zn yarn battery with pristine, 80^°^, 150^°^ of bending strain, and released state. (**b**) Ag-Zn yarn battery within single yarn battery (black line), two serial connected yarn battery (red line), and two parallel connected yarn battery (blue line). (**c**) Photographs of commercial electric watch operated by two serial connected Ag-Zn yarn battery woven in textile watch strap (inset: photographs of yarn electrodes woven in textile watch strap).

One of remarkable advantages of our biscrolled yarn battery is that it is mechanically strong and flexible even at high loading of brittle metal nanoparticle guest loadings. To check the flexibility, the solid-state yarn battery (that comprises of three plied Ag-Zn biscrolled electrodes) was bent at 80 and 150 degree angle, and was recovered to the pristine state during galvanostatic discharge process (Fig. 4a). During the dynamically applied bending deformations, the yarn battery exhibited stable discharge plateaus at 1.4 V with 0.5 mA/cm current density. The two biscrolled yarn batteries were series or parallel connected (Fig. 4b). From the discharge curves, doubled voltage increase by serial connection and doubled capacity increase by parallel connection were observed. Due to the effective connection, the two-series connected solid-state battery could light up a green LED (Fig. S11). Moreover, to demonstrate the possibility of wearable energy storage application, 5 cm long, two series connected, biscrolled Ag-Zn battery yarns were sewn into a watch strap textile and were electrically connected to a commercial electric watch using Cu wire (Fig. S12). Due to high mechanical strength, the arrays of the original fibers in the textile were successfully replaced by the biscrolled yarn electrodes. The electric watch was fully powered and operated well by the woven yarn batteries (Fig. 4d).

## Conclusion

In summary, we developed high performance and safe Ag-Zn battery using active material embedded yarn electrodes and water based electrolyte. Biscrolling method enables the wt% of active materials loaded on Ag yarn electrode up to 99.1 wt% and Zn yarn electrode up to 98.4 wt%. These high loaded Ag and Zn yarn electrodes result in high linear capacity of 0.285 mAh/cm in liquid electrolyte and 0.276 mAh/cm in solid gel electrolyte, which are better than previous reported fiber-based battery systems. Moreover, different from previously reported Li-based battery, our yarn battery is also inflammable, non-explosive and safe due to its aqueous-based PVA-KOH electrolyte. Therefore, our high-capacity Ag-Zn yarn battery can be alternative of energy source for wearable and portable electronics.

## Method

### Chemicals and materials

Due to its high mechanical (144 MPa cm^3^/g) and electrical properties, CNT aerogel sheet ribbon stacks was used as a host of yarn electrode. Well-aligned MWNT forest (~400 um high and consisting of ~12 nm diameter nanotubes containing ~9 walls) was synthesized on a Si wafer using previously reported chemical vapor deposition (CVD) method^38^. Commercially available Ag nanowire and Zn nanoparticle were used as energy storage functional guest materials. Silver nanowires with 115 nm of diameter and 20~50 μm of length dispersed in isopropyl alcohol suspension, zinc nanoparticles with ~50 nm, PVA (average M_w_ is 130,000), and zinc oxide were purchased from Sigma-Aldrich Corporation. Potassium hydroxide was from J. T. Baker.

### Fabrication of Ag-Zn yarn battery and liquid state electrolyte

The fabrication of our high wt% active material loaded yarn battery electrodes was similar to previously reported^31^. Three layers of 120 mm (length) x 15 mm (width) sized CNT sheets drawn from the CNT forest were stacked on the glass substrate. The weight of the stacked MWNT sheets were approximately 11 to 16 μg. To fabricate silver cathode, 200 mL of silver nanowire dispersed in isopropyl alcohol suspension was dropped on the CNT sheet and dried 5 min in room temperature. After repeated drop casting and drying, one end of each stacked CNT sheet with active materials was connected to the electrical motor and twisted about 1500 turns per meter. To fabricate zinc anode, zinc nanoparticles were dispersed in ethanol and high-sonicated for 2 hours. After drop casting on CNT sheet, one end of CNT sheet with active materials was connected to the electrical motor and twisted about 2000 turns per meter. KOH liquid solution was prepared using 33.67 g KOH in 100 mL deionized water. The solution was stirred at 60 turns per minute until KOH particles are dissolved completely. Prepared Ag yarn cathode and Zn yarn anode were fixed to glass slide, so that the yarn electrodes were parallel and closely adjacent. Cu wires with 180 μm diameter were attached to an end of each yarn using silver paste for electrochemical performance characterization, and then the interconnections between yarn electrode and Cu wire were coated by epoxy. The two parallel yarn electrodes were dipped into the 6 M KOH liquid solution.

### Preparation of yarn battery in aqueous-based solid gel electrolyte

KOH + PVA solid gel electrolyte was prepared using 3.37 g KOH and 2 g PVA in 20 mL deionized water. The solution was stirred at 60 turns per minute at 140 °C until it became translucent. Two Ag yarn cathodes and one Zn yarn anode were fixed to glass slide, so that the yarn electrodes were parallel and closely adjacent. Cu wires with 180 μm diameter were attached to an end of each yarn using silver paste for electrochemical performance characterization, and then the interconnections between yarn electrode and Cu wire were coated by epoxy. In order to prevent electrical shortage, 10 wt% PVA was coated on the Zn anode and dried at 60 °C. After drying, three electrodes are plied. The 3-plied yarn battery was completed by coating with 3 M KOH + PVA solid gel electrolyte.

### Morphology analysis and electrochemical performance characterization

The length and weight of the yarn electrodes were measured using a digital Vernier calipers (500 series, Mitutoyo) and micro-balance (XP6, Meter toledo), respectively. SEM images of yarn battery were obtained by scanning electron microscopy (Hitachi S4700). All electrochemical measurements of the yarn battery utilized the electrochemical analyzer, CHI 627b system (CH instruments, Austin, TX). For cross-section analysis, the Ag/CNT hybrid yarn was cut and polished along its diameter using Ga ions in a Focused Ion Beam (FIB NOVA 200). Microstructural and chemical analyses were carried out at scanning electron microscope (SEM) coupled with energy dispersive spectroscopy (Zeiss SUPRA 40 Gemini EDAX). The Zn/CNT hybrid yarn was cut and polished along its diameter using Ga ions in a Focused Ion Beam (FIB NOVA 200). Microstructural and chemical analyses were carried out at Zeiss SUPRA 40 Gemini EDAX and Zeiss-LEO Model 1530. Samples were coated by sputtering with gold for imaging purposes. The porosity of the cross-section of the hybrid yarn was analyzed by a tool for image-based quantitative digital analysis. **Calculation of wt% and length capacity**. The wt% of yarn electrode was calculated using

6 $$wt \% =\frac{{W}_{T}-{W}_{CNT}}{{W}_{T}}\times 100$$

where wt% is weight% of active material, *W*_*T*_ is the total weight of yarn electrode (g), and *W*_*CNT*_ is weight of pure MWNT yarn (g). The length capacity (mAh/cm) was calculated from the Galvanostatic discharging curve using

7 $$Capacity=\frac{{I}\times \Delta t}{Unit}$$

where *I* is the discharging current, *Δt* is the discharging time and *Unit* is the total length, area, volume, or mass of the yarn electrodes.

## Electronic supplementary material

Supplementary Information
