# Supplementary material for: Biscrolled Carbon Nanotube Yarn Structured Silver-Zinc Battery
Source: Sci Rep. 2018 Jul 24;8:11150. doi: 10.1038/s41598-018-29266-0 (PMC6057988; doi:10.1038/s41598-018-29266-0)
Supplement: Supplementary file 1 — Supplementary Information [file 41598_2018_29266_MOESM1_ESM.docx]

Supplementary Information

Biscrolled Carbon Nanotube Yarn Structured Silver-Zinc Battery

**Jae Myeong Lee^1,+^, Changsoon Choi^2,^**^+^**, Ji Hwan Kim^1^, Mônica Jung de Andrade^3^, Ray H. Baughman^3^ and Seon Jeong Kim^1,^***

^1^ *Center for Self-powered Actuation, Department of Biomedical Engineering, Hanyang University, Seoul 04763, Korea*

^2^ *Division of Smart Textile Convergence Research, Daegu Gyeongbuk Institute of Science and Technology (DGIST), Daegu 42988, Korea*

^3^ *The Alan G. MacDiarmid NanoTech Institute, University of Texas at Dallas, Richardson, TX 75083, USA*

* To whom correspondence should be addressed. E-mail: [sjk@hanyang.ac.kr](mailto:sjk@hanyang.ac.kr)

^+^ These authors are equally contributed to this work.

**
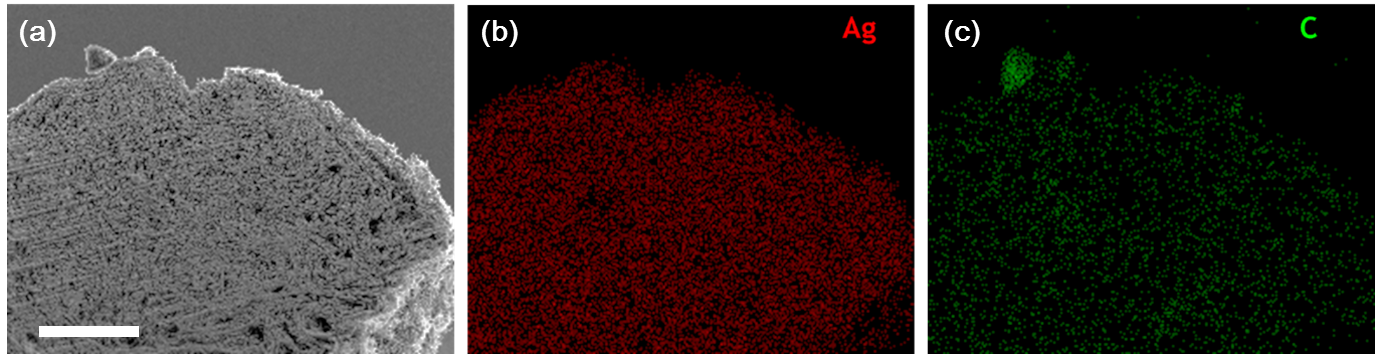
**


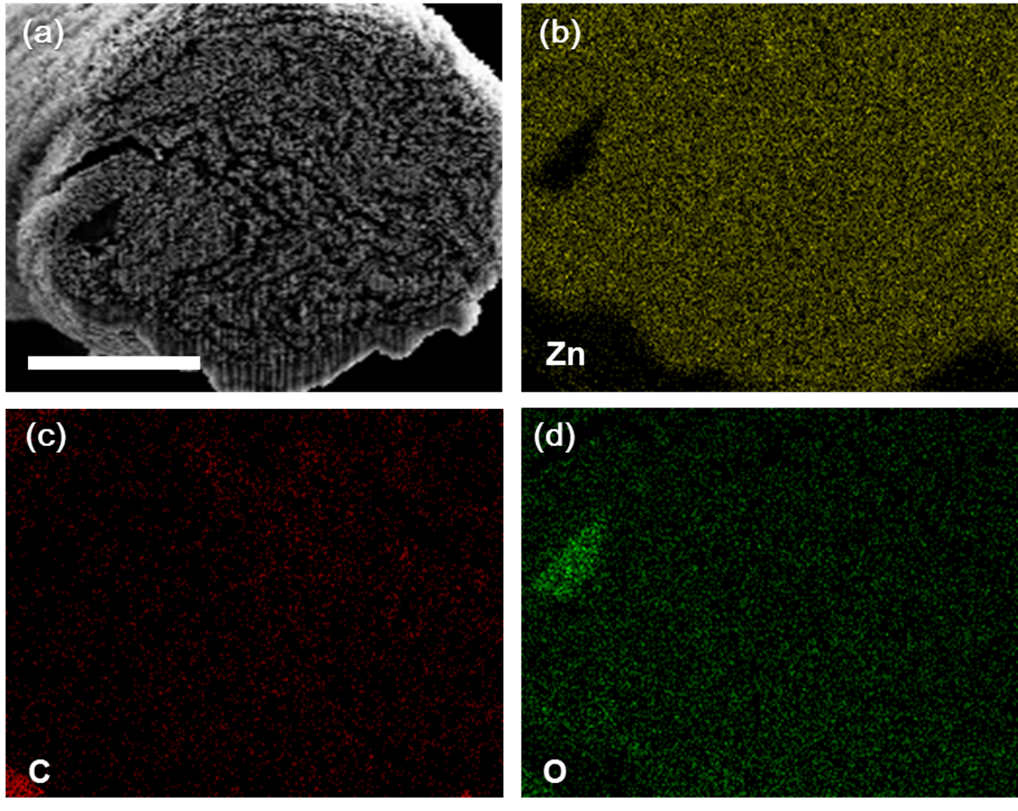
**Figure S1.** (a) SEM micrographs of the Ag/CNT hybrid yarn cross-section. (b) EDX mapping for Ag and (c) C elements showing a homogeneous distribution of the Ag guest into the CNT host (scale bar = 20 μm).

**Figure S2.** (a) SEM micrographs of the Zn/CNT hybrid yarn cross-section. (b) EDX mapping for Zn, (c) C, and (d) O elements showing a homogeneous distribution of the Zn guest into the CNT host (scale bar = 30 μm).


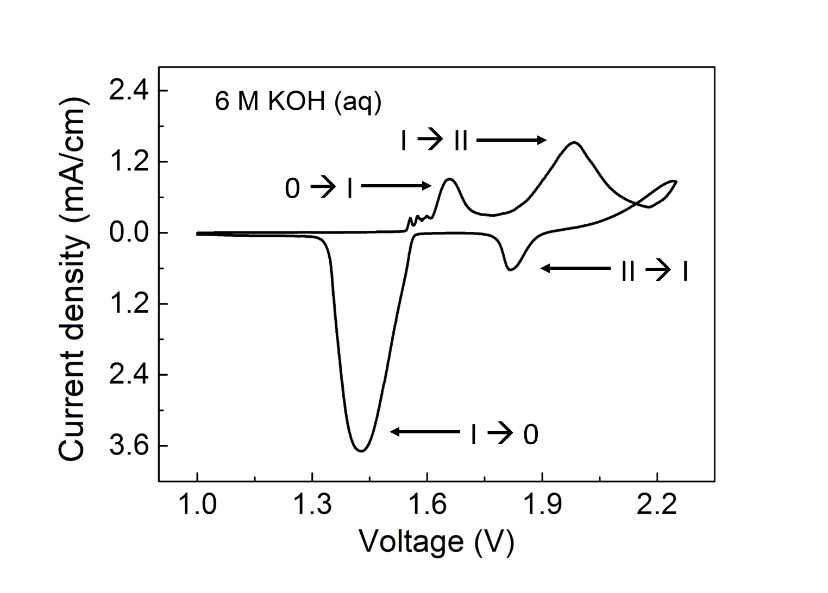

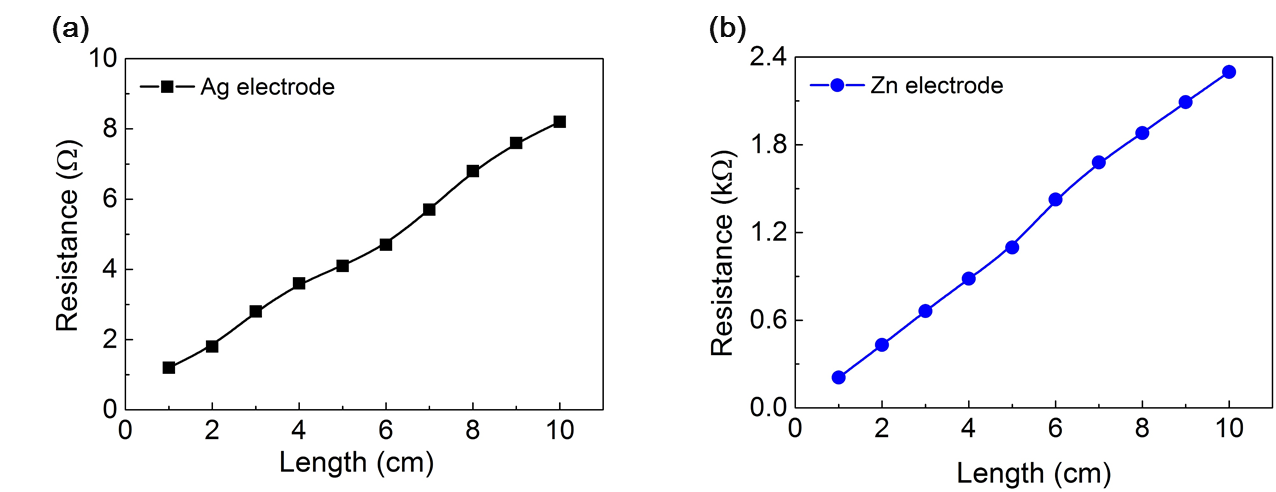
 **Figure S3.** Resistance of the (a) Ag yarn electrode (diameter=344.1 µm) and (b) zinc electrode (diameter=239.2 µm) as a function of length.

**Figure S4.** CV curve of Ag-Zn yarn battery in 6 M KOH liquid solution measured at scan rate of 10 mV/s. Zn anode was used as a counter and reference electrode.


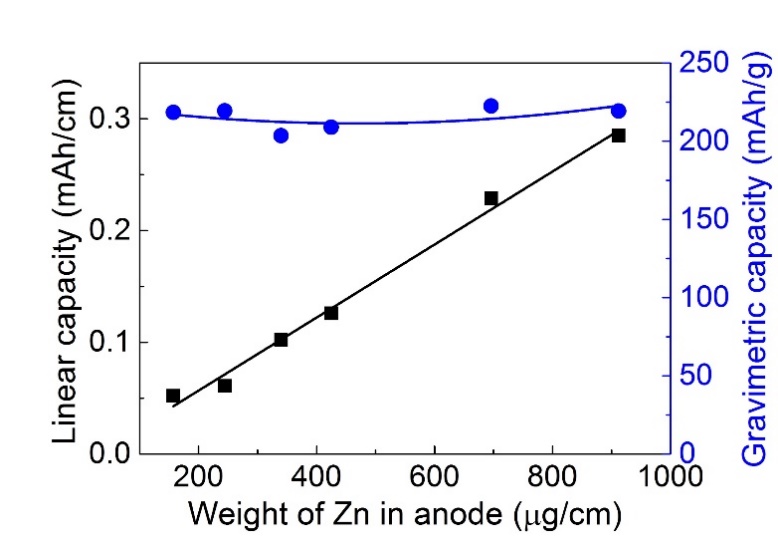


**Figure S5.** Linear and gravimetric capacities as a function of weight of Zn in anode.

**
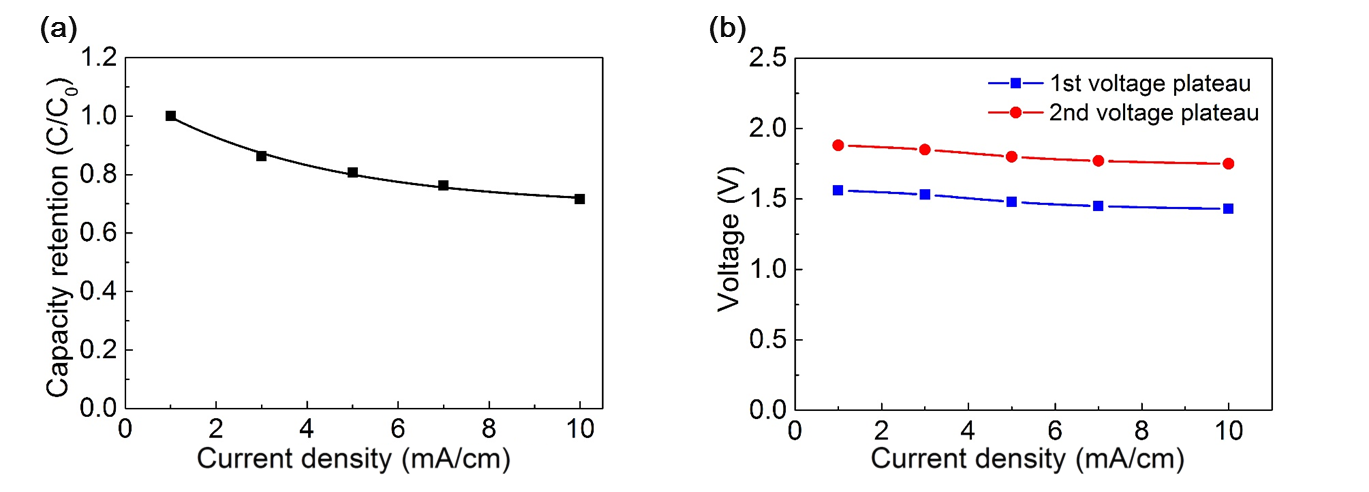
**

**Figure S6.** (a) Capacity retention and (b) voltage changes of silver-zinc yarn battery versus current densities of 1 to 10 mA/cm.


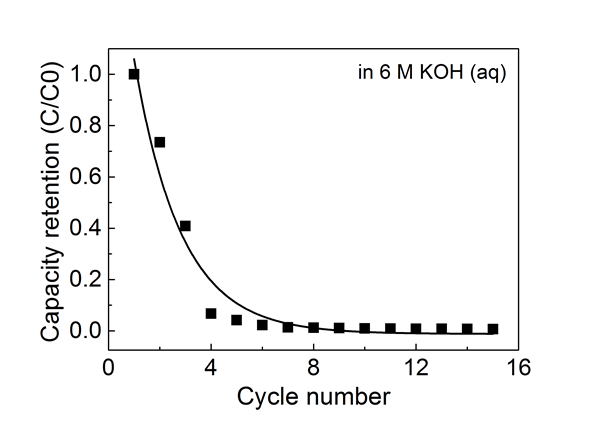


**Figure S7.** Capacity retention during repeated 50 cycles of charge-discharge in 6 M KOH liquid solution.

**
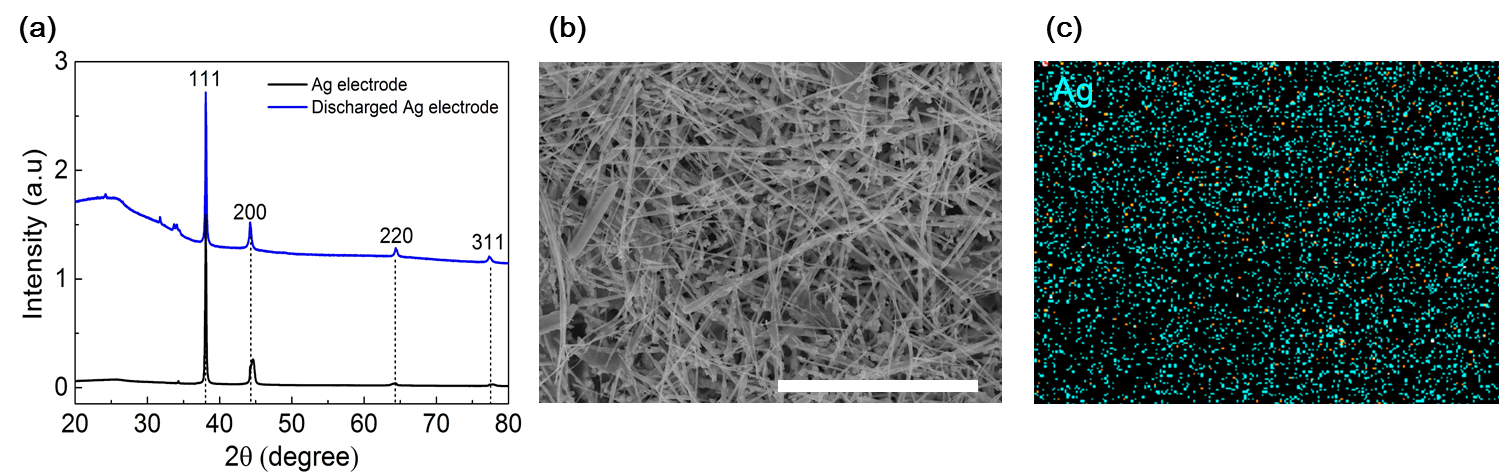
**

**Figure S8.** (a) XRD patterns of Ag cathode before and after 20 cycles of discharging. (b) SEM image (scale bar = 10 μm) and (c) EDX mapping of Ag electrode after 20 cycles of discharging.

**
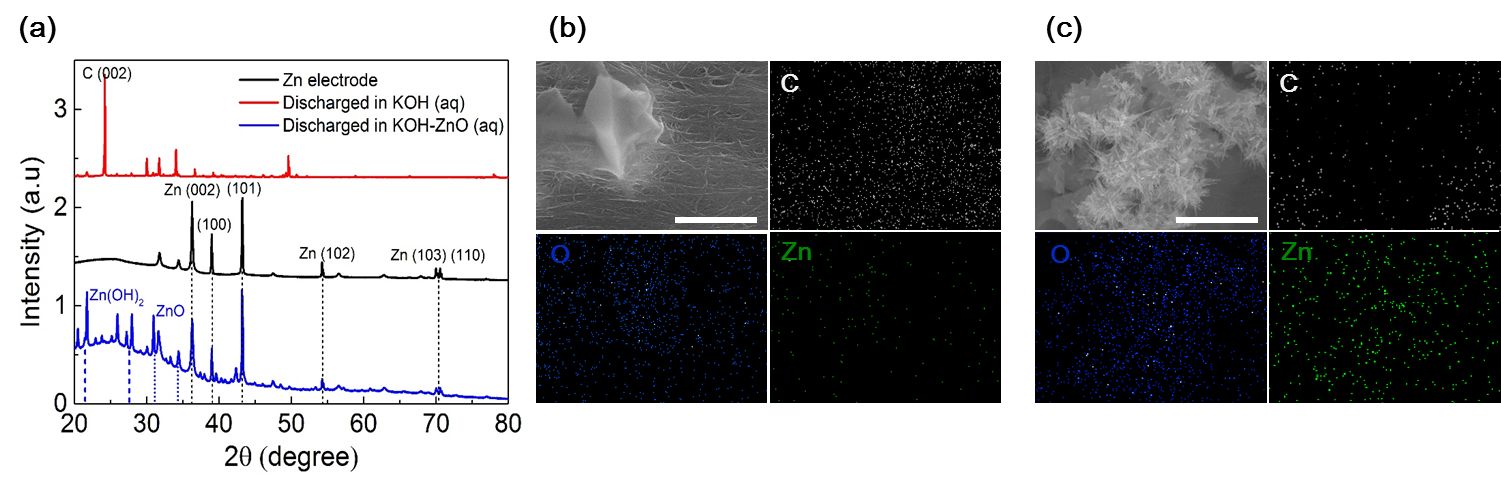
**

**Figure S9.** (a) XRD patterns of Zn anode before (black line) and after 20 cycles of discharging in KOH electrolyte (red line), and 20 cycles of discharging in KOH + ZnO electrolyte (blue line). SEM image and EDX mapping of Zn electrode after 20 cycles of discharging in (b) KOH electrolyte, and (c) KOH + ZnO electrolyte (scale bar = 3 μm).

**
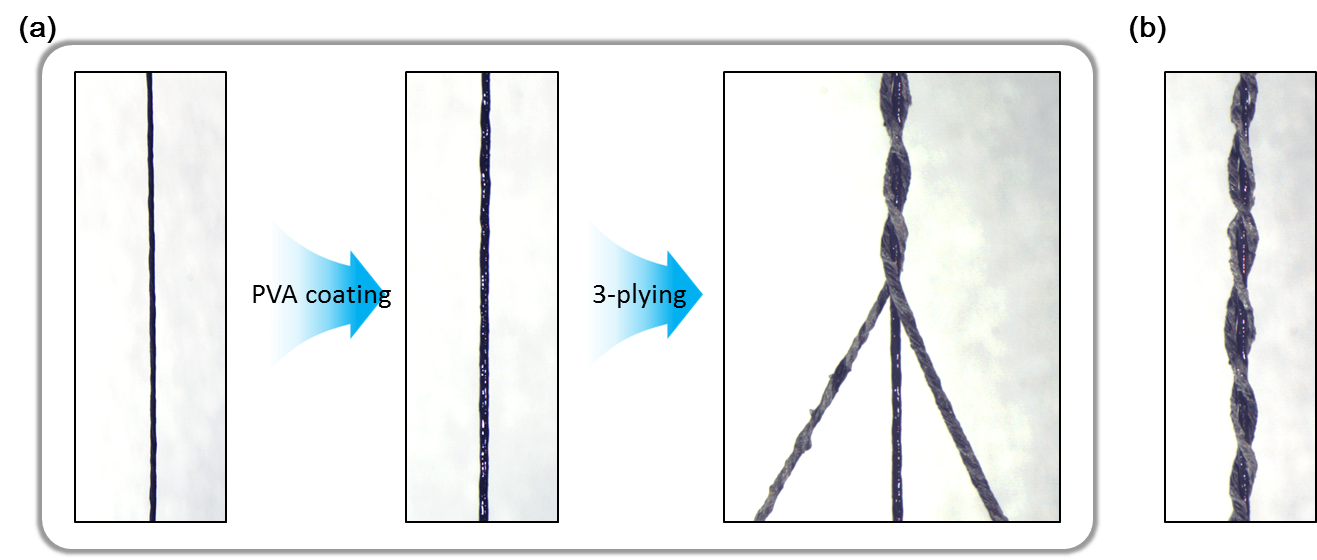
**

**Figure S10.** (a) Fabrication processes of 3-plied silver-zinc yarn battery, (b) 3-plied silver-zinc yarn biscrolling battery.


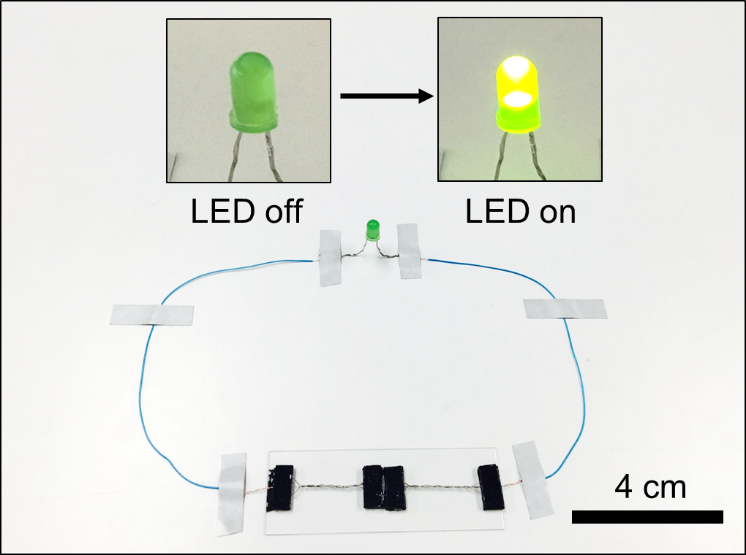


**Figure S11.** LED circuit with serial connection of two batteries (inset: photographs of unlighted and lighted green LED by Ag-Zn yarn batteries).

**
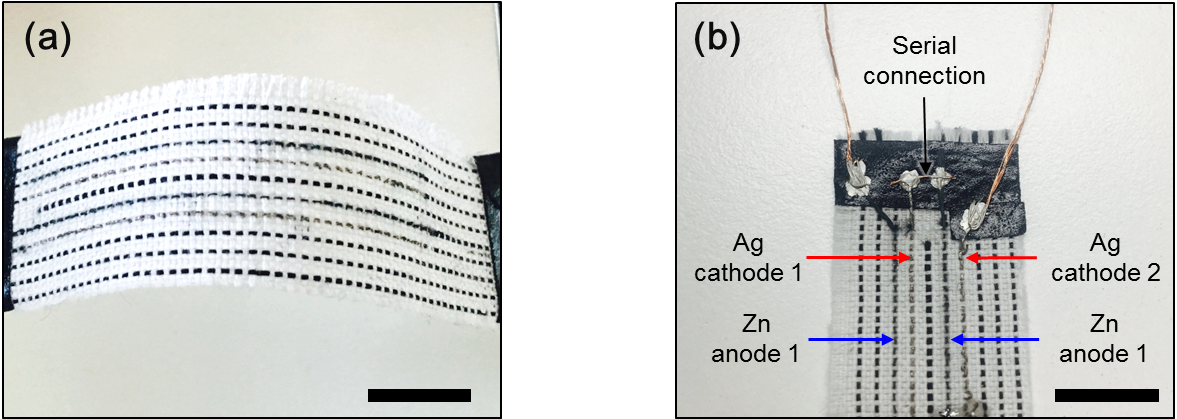
**

**Figure S12.** Photographs of (a) two serial connected Ag-Zn yarn batteries woven in textile watch strap (scale bar = 1.5 cm) and (b) the reverse side of battery woven watch strap (scale bar = 1 cm).
